# Generation of Live Piglets from Cryopreserved Oocytes for the First Time Using a Defined System for *In Vitro* Embryo Production

**DOI:** 10.1371/journal.pone.0097731

**Published:** 2014-05-20

**Authors:** Tamás Somfai, Koji Yoshioka, Fuminori Tanihara, Hiroyuki Kaneko, Junko Noguchi, Naomi Kashiwazaki, Takashi Nagai, Kazuhiro Kikuchi

**Affiliations:** 1 Animal Breeding and Reproduction Research Division, NARO Institute of Livestock and Grassland Science, Tsukuba, Ibaraki, Japan; 2 Pathology and Pathophysiology Research Division, National Institute of Animal Health, Tsukuba, Ibaraki, Japan; 3 Animal Development and Differentiation Research Unit, National Institute of Agrobiological Sciences, Tsukuba, Ibaraki, Japan; 4 The United Graduate School of Veterinary Science, Yamaguchi University, Yamaguchi, Yamaguchi, Japan; 5 Graduate School of Veterinary Sciences, Azabu University, Sagamihara, Kanagawa, Japan; 6 Food and Fertilizer Technology Center, Taipei, Taiwan; Institute of Zoology, Chinese Academy of Sciences, China

## Abstract

We report the successful piglet production from cryopreserved oocytes for the first time by using a simple, high capacity vitrification protocol for preservation and a defined system for *in vitro* embryo production. Immature cumulus-oocyte complexes (COCs) from prepubertal gilts were vitrified in microdrops and stored in liquid nitrogen. After warming, COCs were subjected to *in vitro* maturation (IVM), fertilization (IVF), and subsequent culture (IVC). Adjusting warmplate temperature to 42°C during warming prevented temperature drops in a medium below 34.0°C and significantly increased the percentage of oocyte survival and thus blastocyst yields obtained from total vitrified oocytes compared with that of warming at 38°C (87.1% vs 66.9% and 4.4% vs 2.7%, respectively). Nuclear maturation and fertilization of oocytes were not affected by vitrification and warming temperature. Blastocyst development on day 7 (day 0 = IVF) of the surviving oocytes after warming at 38°C and 42°C was not different but lower (*P*<0.05) than those of non-vitrified control oocytes (4.6%, 5.2% and 17.9%, respectively). However, blastocyst cell numbers in the control and vitrified groups were similar irrespective of warming temperature. Omitting porcine follicular fluid (pFF) from IVM medium (POM) did not affect maturation, fertilization and embryo development of vitrified-warmed oocytes. Transfer of blastocysts obtained on day 5 from vitrified oocytes matured either with or without pFF into 4 recipients (2 for each group) resulted in 4 pregnancies and the delivery of a total of 18 piglets. In conclusion, optimization of warming temperature was a key factor for achieving high survival rates, and surviving oocytes could be utilized *in vitro* using defined media. Using these modifications, live piglets could be obtained from cryopreserved oocytes for the first time.

## Introduction

Oocyte cryopreservation has a strategic importance for the gene baking of female resources serving the preservation of genetic diversity and also for the distribution of genetic lines. However, porcine oocytes have been known to be extremely sensitive to cryopreservation in comparison with oocytes of other species [1]. Although several vitrification protocols have been applied for porcine oocytes with various survival rates, they have been associated with severely compromised embryo development [2]. Vitrification of matured porcine oocytes has been characterized by high survival rates; however, production rates and total yields of blastocyst stage embryos by in vitro fertilization (IVF) or intracytoplasmic sperm injection (ICSI) have remained very low [2]. Despite of their high survival rates, porcine oocytes vitrified at the MII stage often show spindle abnormalities, the accumulation of reactive oxygen species and parthenogenetic activation caused by the cooling/warming process, which greatly reduce their fertilization and developmental competence [3], [4]. For this reason, production of live offspring from cryopreserved porcine oocytes has not been reported to date.

We have already demonstrated that, despite of their low survival rates, porcine oocytes vitrified at the immature germinal vesicle (GV) stage have the ability to undergo nuclear and cytoplasmic maturation during in vitro maturation (IVM) and, after IVF, they can develop to blastocysts with high quality by in vitro culture (IVC) [5]. Nevertheless, in this case, low (approximately 25%) survival rates greatly limited the numbers of blastocyst stage embryos that could be potentially used for embryo transfer (ET). Recently, we have improved our vitrification protocol by customizing the cryoprotectant regimen specifically for porcine oocytes at the GV stage which resulted in reasonable (approximately 50%) survival rates and enabled a relatively stable blastocyst production by subsequent IVM, IVF and IVC [6]. Vitrification of porcine oocytes at the GV-stage by this method was effective to circumvent the problems of spindle damage and parthenogenesis caused by vitrification at the MII stage [4]. The question remains, however, as to whether embryos obtained from vitrified oocytes can develop to term.

Our cryopreservation method is Solid Surface Vitrification (SSV), modified from the original protocol of Dinnyes et al. [7], and previously, it has resulted in the production of live piglets by the cryopreservation of in vitro produced zygotes for the first time [8]. This method enables the preservation of approximately total 100 oocytes in 4−6 microdrops (about 15−25/microdrop) for a single vitrification procedure. During the warming procedure, these microdrops retrieved from liquid nitrogen are dropped simply in a common culture dish containing the warming medium pre-warmed to 38°C. Consecutive insertion of several vitrified objects in warming media may, however, gradually reduce the medium temperature and thus the warming speed. During the warming of vitrified embryos or oocytes, intracellular ice can form around −80°C which is detrimental for their viability [9]. Recently it has been confirmed in mice that high-and-stable warming rates are crucial to pass through this dangerous temperature zone and are essential to provide high survival rates for oocyte vitrification [10]–[12]. The actual temperature of the warming medium is an important factor determining the warming speed. We anticipated that during warming vitrified oocytes, keeping the warmplate at a temperature slightly higher (such as 42°C) than the physiological optimum temperature (38°C) may be effective to prevent extreme drops temperature of the warming medium and thus to provide high and stable survival rates of vitrified porcine oocytes, when several vitrified microdrops are consecutively introduced to a common warming dish.

Besides the actual vitrification and warming protocols, the medium used for in vitro maturation (IVM) after warming also affects the ability of vitrified immature stage porcine oocytes to undergo nuclear maturation and to reach the metaphase-II (M-II) stage [13]. Our recent results have demonstrated that when simple media such as North Carolina State University (NCSU)-37 [14] is used for IVM, supplementation with 10% (v/v) pig follicular fluid (pFF) is essential for the vitrified oocytes to reach the M-II stage [13]. Nevertheless, application of a more complex medium such as the porcine oocyte medium (POM) [15] provides high maturation rates irrespective of pFF supplementation during IVM of vitrified oocytes [13]. The use of chemically defined media is expedient for in vitro embryo production (IVP) systems in order to prevent the uncontrollable effects of undefined factors including pathogens such as viruses which are present in pFF. However, the fertilization and developmental competence of vitrified oocytes after IVM either in pFF free or enriched POM medium has remained unknown.

Our objectives were to 1) improve the efficacy of our vitrification protocol, especially by controlling warming temperature, in terms of survival rates and thus the yield of transferable embryos by IVP, 2) to employ completely defined media for embryo production and 3) to produce live piglets using cryopreserved oocytes for the first time. To achieve these goals, we studied the effects of the temperature of the warmplate and the medium during warming and the presence/absence of pFF during IVM on survival rates, nuclear maturation, in vitro fertilization and subsequent in vitro embryo development. Finally, blastocyst stage embryos derived from vitrified immature stage porcine oocytes were transferred to recipients to test their ability to develop to term.

## Materials and Methods

All chemicals were purchased from the Sigma-Aldrich Corporation (St. Louis, MO, USA), unless otherwise indicated.

### Ethics Statement

Protocols for the use of animals were approved by the Institutional Care and Use Committee of the National Institute of Animal Health, Japan (Protocol No. 12-083).

### Collection and Vitrification of Immature Oocytes

Collection of porcine follicular oocytes was performed according to the method of Kikuchi et al. [16]. Ovaries from prepubertal cross-bred gilts (Landrace, Large White, and Duroc breeds) were collected at a local slaughterhouse and transported to the laboratory in Dulbecco’s phosphate-buffered saline (PBS) (Nissui Pharmaceutical Co. Ltd, Tokyo, Japan) at 35 to 37°C within 1 h. Cumulus–oocyte complexes (COCs) were collected by scraping of 3- to 6-mm follicles into a collection medium consisting of Medium 199 (with Hanks’ salts) supplemented with 5% (v/v) fetal bovine serum (Gibco; Invitrogen Corp., Carlsbad, CA, USA), 20 mM HEPES (Dojindo Laboratories, Kumamoto, Japan), and antibiotics [100 units/ml penicillin G potassium and 0.1 mg/ml streptomycin sulfate]. The COCs were cryopreserved as reported previously [6], with slight modifications. In brief, COCs were treated for 30 min in a basic medium (BM) consisting of modified NCSU-37 [14] without glucose, but supplemented with 20 mM HEPES, 50 µM β-mercaptoethanol, 0.17 mM sodium pyruvate, 2.73 mM sodium lactate. The BM medium was further supplemented with 4 mg/ml bovine serum albumin (BSA; Fraction V) and 7.5 µg/ml cytochalasin B (C-6762) for the first 30 min treatment before equilibration. The COCs were then treated with an equilibration medium, which was BM supplemented with 2% (v/v), ethylene glycol (EG, E-9129), 2% (v/v) propylene glycol (PG, Nacalai Tesque Inc., Kyoto, Japan, 29218-35), 7.5 µg/ml cytochalasin B, and 4 mg/ml BSA. The COCs were incubated in the equilibration medium for 13−15 min at 38.5°C, washed three times in 20-µl drops of vitrification solution at 38.5°C, then pipetted into a glass capillary tube in groups of 25 to 30, and finally, in about 2–3 µL of vitrification solution, dropped onto aluminum foil floating on the surface of liquid nitrogen (LN). The vitrification solution was BM supplemented with 50 mg/ml, polyvinylpyrrolidone (P-0930), 0.3 M trehalose (T-0167), 17.5% (v/v) EG, 17.5% (v/v) PG and 4 mg/ml BSA. Washing in vitrification medium and placing microdrops with COCs onto the cold aluminum surface were performed within about 30 s in total. The vitrified droplets were transferred to 2-ml cryotubes (Iwaki 2732-002; AGC Techno Glass Co. Ltd., Tokyo, Japan) partly immersed in LN, and then stored in LN for several weeks until use. Before the start of the warming procedure, the warming medium (kept airtight in 15 ml centrifuge tubes) was pre-warmed to 38°C for 30 minutes in an incubator, then kept at 38°C or 42°C in a dry block tube heater fitted on a warmplate (SP-45D, Hirasawa, Tokyo, Japan) for an additional 20 minutes according to experimental design (detailed below). Vitrified droplets were warmed by transfer into 2.5 ml of a warming solution (0.4 M trehalose in BM, supplemented with 4 mg/ml BSA) in a 35-mm plastic dish (Falcon 351008, Becton Dickinson Labware, NJ, USA) kept on a warmplate kept at either 38°C or 42°C according to experimental design (detailed below). One to two minutes later, oocytes were consecutively transferred for periods of 1 min (each) to 500-µl droplets of BM supplemented with 0.2, 0.1 or 0.05 M Trehalose at 38.0°C. They were then washed in BM without trehalose at 38.0°C and subjected to IVM. During the warming process, the actual inner temperature of the tube heater and the surface temperature of the warmplate were verified both by the built-in thermometer of the warmplate and an analog thermometer.

### *In Vitro* Maturation (IVM)

The basic maturation culture medium was POM [15] (Cat no. IFP1010P, Research Institute for the Functional Peptites, Higashine, Japan) supplemented with or without 10% (v/v) pFF, according to experimental design (detailed below). The pFF was collected in advance by aspiration with a syringe and centrifuged at 1800×*g* for 1.5 h and the supernatant was stored at −20°C. Then about 1 l of the stock was thawed, mixed, centrifuged again, sorted out in 10 ml aliquots and stored at −20°C as a single batch until use. During the first 22 h of IVM, the medium was supplemented with 1 mM dibutyryl cAMP (dbcAMP), 10 IU/ml eCG (Serotropin; ASKA Pharmaceutical Co. Ltd., Tokyo, Japan), and 10 IU/ml hCG (500 units; Puberogen, Novartis Animal Health, Tokyo, Japan) as well. Maturation was performed in 4-well dishes (Nunclon Multidishes, Nalge Nunc International, Roskilde, Denmark) in 500-µl droplets of IVM medium without a paraffin oil covering in an atmosphere of 5% CO_2_, 5% O_2_, and 90% N_2_ at 39°C. The COCs were subsequently cultured in the maturation medium without dbcAMP and hormones for an additional 22 h under the same atmosphere. Forty to sixty COCs were cultured in each well.

### Evaluation of Live/Dead Status and Nuclear Maturation of IVM Oocytes

At the end of IVM, COCs were transferred for 30 sec to 1 ml of collection medium supplemented with 0.1% (w/v) hyaluronidase and gently pipetted to remove the cumulus cells. The oocytes were washed twice in a hyaluronidase-free collection medium and observed under a stereo microscope. Oocytes with clear signs of membrane damage (brownish, faded cytoplasm) were defined as dead, and removed. Only oocytes with a normal spherical shape, smooth surface, and a dark and evenly granuled cytoplasm were considered viable. Oocytes selected by this criteria have been proven to be functionally alive using fluorescein diacetate staining [5]. Live oocytes with a visible first polar body (1PB; the indicator of the metaphase II stage) were considered to be matured.

### *In Vitro* Fertilization (IVF) of Oocytes and In Vitro Culture (IVC) of Embryos

The IVF and IVC procedures were performed as reported previously [16]. The medium for IVF was a modified Pig Fertilization Medium [17], which consisted of 90 mM NaCl, 12 mM KCl, 25 mM NaHCO_3_, 0.5 mM NaH_2_PO_4_, 0.5 mM MgSO_4_, 10 mM sodium lactate, 10 mM HEPES, 8 mM CaCl_2_, 2 mM sodium pyruvate, 2 mM caffeine, and 5 mg/ml bovine serum albumin (BSA; Fraction V). After being washed three times in IVF medium, oocytes were transferred into 100-µl IVF droplets in 35-mm plastic dishes (Falcon 351008) covered by paraffin oil (Paraffin Liquid; Nacalai Tesque, Inc., Kyoto, Japan). About 20 oocytes were transferred in each droplet. In order to reduce the variation of fertilization results caused by sperm source, Landrace epididymal sperm frozen as a single lot in 0.25 ml straws [18] was used throughout the study. After thawing in a 38.0°C waterbath, spermatozoa were centrifuged in 7 ml of a pre-warmed Medium 199 (with Earle’s salts, Gibco, pH adjusted to 7.8) for 2 min at 750×*g.* Then, the pellet was re-suspended in 70 µl of the same medium, covered by paraffin oil and preincubated in for 15 min [18]. A small portion of sperm suspension was introduced into the IVF medium containing oocytes and coincubated for 3 h at 39°C under 5% CO_2_, 5% O_2_, and 90% N_2_. The final sperm concentration was 1.5×10^5^ cells/ml (previously determined as the optimum concentration for the sperm lot used). The day of IVF was defined as day 0. After removal of spermatozoa from the surface of the zona pellucida by gentle passage through a fine glass pipette, IVC was performed in 500-µl drops of the chemically defined porcine zygote medium 5 [15] (PZM-5; Cat no. IFP0401P, Research Institute for the Functional Peptites) without oil covering for days 0 to 7 in 4-well dishes under an atmosphere of 5% CO_2_, 5% O_2_, and 90% N_2_ at 39°C. Cleavage rates were recorded on day 2. Blastocysts were harvested on days 5, 6 and 7. In the present study, blastocysts harvested on day 5 were used either for the evaluation of embryo quality (measured by total cell numbers) or ET, according to experimental design. Blastocysts harvested on days 6 and 7, on the other hand, were used for cell number evaluation.

### Evaluation of Fertilization Status

The fertilization status of oocytes was assessed 10 h after IVF. Oocytes were mounted on glass slides and fixed with acetic alcohol (1∶3 acetic acid:ethanol) for at least 3 days, then stained with 1% (w/v) orcein in acetic acid, rinsed in glycerol:acetic acid:water (1∶1∶3) and examined under a phase-contrast microscope with ×40 and ×100 objectives. The presence and numbers of female and male pronuclei and/or a sperm head(s), and extrusion of the two bolar bodies, were then investigated. An oocyte was considered to be activated if a female pronucleus was detected in the cytoplasm. Oocytes were considered to have been penetrated when a sperm head(s) or a male pronucleus(ei) with the corresponding sperm tail(s) were detected in the cytoplasm. Oocytes with a female pronucleus but without a penetrating sperm were considered to have been activated parthenogenetically. Normal fertilization was defined by the presence of one female pronucleus and one male pronucleus, and the extrusion of both the 1st and 2nd polar bodies. Oocytes with one penetrating sperm in the cytoplasm were defined as monospermic.

### Evaluation of Blastocyst Quality

The quality of in vitro produced embryos was measured by evaluating total cell numbers in blastocysts on day 7. Blastocysts harvested on day 5 and day 6 were consecutively cultured in a porcine blastocyst medium [19] (PBM; Cat no. IFP1030P, Research Institutes for the Functional Peptides) to day 7 to prevent their premature collapse. These embryos along with newly developed blastocysts on day 7 were subjected to nuclear staining. To assess the total numbers of cells in embryos, blastocysts were simultaneously treated with 25 µg/ml Hoechst 33342 (H33342, Calbiochem, San Diego, CA, USA) dissolved in 99.5% ethanol overnight. After washing in 99.5% ethanol they were mounted on glass slides in glycerol droplets, flattened by cover slips, and examined under UV light with an excitation wavelength of 330–385 nm using an epifluorescence microscope (IX-71, Olympus, Tokyo, Japan). A digital image of each embryo was taken, and the total numbers of nuclei labeled by H33342 were counted using the NIH Image J (v.1.40) software package [20].

### Embryo Transfer

Estrus synchronization of the recipients was achieved according to a previous report [21]. In brief, cross bred gilts (Landrace×Large White, 7 mo old, 95–101 kg body weight) received intramuscular injections of Prostaglandin F2α, as 15 mg Dinoprost (Panacelan Hi; Meiji Seika, Tokyo, Japan) twice a day in a 3-day period during the functional luteal phase to induce luteolysis. At the fifth administration of prostaglandin F_2α_, the gilts were injected with 1000 IU of eCG intramuscularly, followed 72 h later by 750 IU of hCG to induce ovulation. Day-5 embryos that developed to the blastocyst stage were surgically transferred into the uteri of 4 recipients (13–27 blastocysts/recipient) 5 days after hCG injections. Pregnancy diagnosis was carried out by ultrasonography 25 days after hCG injections. Pregnant gilts were allowed to continue to full term. Gestation lengths, litter sizes and body weights of the piglets at birth were examined.

### Experimental Design

#### Experiment 1: Effect of warming temperature on survival, maturation, fertilization and in vitro development of vitrified porcine oocytes

COCs vitrified as the same batch and stored for 2–4 weeks were warmed on a warmplate kept at either 38°C or 42°C for 30 sec. Then, the warming dish was placed to a warmplate heated to 38°C and further procedures were conducted at this temperature. Vitrified COCs warmed either at 38°C or 42°C along with freshly collected non-vitrified COCs (control) were subjected to IVM using a base medium with 10% (v/v) pFF supplementation. Oocyte survival and nuclear maturation were evaluated after IVM. Live oocytes were subjected to IVF and IVC. Fertilization status, embryo development and blastocyst quality were compared among groups. The experiment was replicated five times. In a separate experiment, temperature changes were monitored during consecutive warming of 6 vitrified microdrops of the vitrification solution (each 2 µl) in a common warming dish placed either on a 38°C or a 42°C warmplate using a digital recorder (TNA-140, Tasco Japan Co., Ltd, Osaka, Japan). The temperature of the warming medium in the culture dish was recorded in 2 sec intervals for a 60 sec period. The experiment was replicated three times.

#### Experiment 2: Effect of pFF supplementation in IVM medium on survival, maturation, fertilization and in vitro development of vitrified porcine oocytes

Based on the results of *Experiment 1*, COCs vitrified as the same batch and stored for 2–4 weeks were warmed on a warmplate kept at 42°C for 30 sec. Then, further procedures were conducted at 38°C as described above. The vitrified-warmed COCs were then subjected to IVM with or without 10% (v/v) pFF supplementation in base medium. Oocyte survival and nuclear maturation were evaluated after IVM. Live oocytes were subjected to IVF and IVC. Fertilization status, embryo development and blastocyst quality were compared among groups. Five replications were performed.

#### Experiment 3: Transfer of blastocyst produced from vitrified oocytes to recipients

Vitrified COCs stored for 2–4 weeks in LN were warmed on a 42°C warmplate based on the results of the first experiment. Warmed COCs were then subjected to IVM either in pFF-supplemented or chemically defined POM media. IVF of surviving oocytes and subsequent embryo culture were performed as described above. After in vitro culture for 5 days, embryos at the blastocyst or early blastocyst stage derived from vitrified oocytes after IVM (Figure S1 in File S1) were washed three times with porcine oocyte/embryo collection medium (POE-CM; Cat no. IFP1040P, Research Institutes for the Functional Peptides) and then transferred with the same medium in the uteri of recipients.

### Statistical Analysis

Data of oocyte survival, nuclear maturation, fertilization and subsequent embryo development in *Experiment 1* were analysed by one-way ANOVA followed by Tukey’s multiple comparison test. Data of maximum and minimum medium temperatures during warming in *Experiment 1* and all data of *Experiment 2* (employing only 2 experimental groups) were analysed by student’s t-test. All statistical analyses were performed by KyPlot package (Ver. 4.0; Kyens Laboratory. Inc., Tokyo, Japan). Percentage data were arcsine transformed before analysis. P-values<0.05 were considered significant.

## Results

### Effect of Warming Temperature on Survival, Maturation, Fertilization and *In vitro* Development of Vitrified Porcine Oocytes

The proportion of surviving oocytes in the vitrified group warmed at 42°C (87.1%) was significantly higher than that in the vitrified group warmed at 38°C (66.9%); however, both of these values were significantly lower than that of the control group (99.7%) (Table 1). The percentages of live oocytes reaching the M-II stage were similar among the treatment groups (ranging from 61.7% to 66.8%) (Table 1).

**Table 1:** Survival and nuclear maturation of control and vitrified oocytes at 44

Treatment	Hotplate temperature at warming	Total	Live (% total)	Matured with 1 PB (% live)
Control	–	397	396 (99.7±0.28)^a^	264 (66.8±4.6)
Vitrified	38°C	615	412 (66.9±2.4)^c^	253 (61.7±1.8)
Vitrified	42°C	534	463 (87.1±2.6)^b^	299 (64.3±3.0)

Five replications were performed. Data are presented as means ± SEM.

IVM = in vitro maturation, PB = polar body.

a,b,c Percentages with different letters differ significantly (*P*<0.05) in the same column by one-way ANOVA followed by Tukey’s multiple comparison test.

**Figure 1:**
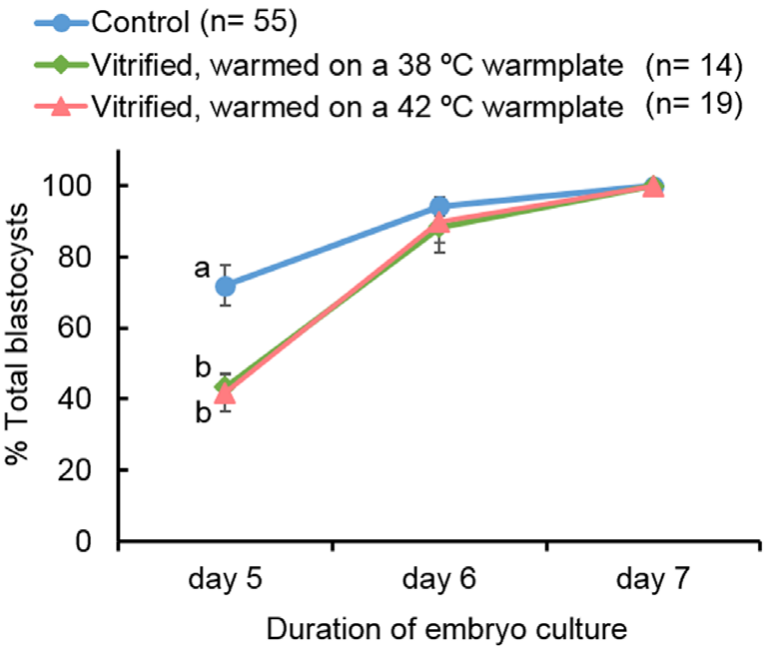
Dynamics of blastocyst formation during *in vitro* culture in PZM-5 medium of embryos derived by IVF from control and vitrified oocytes warmed at different temperatures. Numbers of total blastocyst in each group are given in parentheses. Percentages (± SEM) with different superscripts (a, b) at the same time point differ significantly (*P*<0.05) by one-way ANOVA followed by Tukey’s multiple comparison test.

After IVF of live oocytes, percentage of oocytes showing sperm penetration, male pronuclear formation, monospermy and normal fertilization did not differ significantly among the 3 groups (Table S1 in File S1). During embryo culture, the cleavage rates in vitrified groups warmed at either 38°C or 42°C (42.1%, and 37.3%, respectively) were similar to each another, but significantly lower than that in the control group (65.3%) (Table 2). The percentages of fertilized and cultured oocytes that developed to the blastocyst stage in the vitrified groups warmed at 38°C or 42°C were significantly lower compared with the control group on day 5 (1.9%, 2.2% and 12.9%, respectively), day 6 (3.9%, 4.6% and 16.9%, respectively) and day 7 (4.6%, 5.2% and 17.9%, respectively) (Table 2). When blastocyst formation dynamics were expressed as the percentage of total blastocysts developed during embryo culture, blastocyst development dynamics on day 5 in vitrified groups warmed at either 38°C or 42°C were similar to one another, but significantly lower than that in the control group (43.3%, 41.6%, and 71.9% respectively) (Figure 1). However, on day 6 there was no statistical difference among 3 groups in the percentage of blastocyst formation calculated from total blastocysts (Figure 1). Also, the total number of cells in cultured blastocysts on day 7 among the control and vitrified groups were similar (53.5±4.1, 48.2±2.6 and 50.8±2.2, respectively) (Table 2). When the total blastocyst yield was calculated as the percentage of day 7 blastocysts from the original number of oocytes subjected to vitrification, the vitrified group warmed at 42°C showed a significantly higher blastocyst yield compared with the vitrified group warmed at 38°C (14/615 = 2.7±0.3% and 19/534 = 4.4±0.4%, respectively).

**Table 2:** *In vitro* embryo development after IVM and IVF of control and vitrified oocytes.

Treatment	Hotplate temperatureat warming	Total§	Cleaved (% total)	Blastocyst (% total)			No. of cells/blastocyst
				day 5	day 6	day 7	
Control	–	298	194 (65.3±3.6)^a^	40 (12.9±1.4)^a^	52 (16.9±1.2)^a^	55 (17.9±1.1)^a^	53.5±4.1
Vitrified	38°C	314	133 (42.1±1.2)^b^	6 (1.9±0.4)^b^	12 (3.9±0.5)^b^	14 (4.6±0.8)^b^	48.2±2.6
Vitrified	42°C	365	138 (37.3±4.3)^b^	8 (2.2±0.3)^b^	17 (4.6±0.3)^b^	19 (5.2±0.4)^b^	50.8±2.2

Five replications were performed. Data are presented as means ± SEM.

a,b Percentages with different letters differ significantly (*P*<0.05) in the same column by one-way ANOVA followed by Tukey’s multiple comparison test.

IVM = in vitro maturation, IVF = in vitro fertilization, Day 0 = the day of IVF.

§ Live oocytes at 44 h IVM.

**Figure 2:**
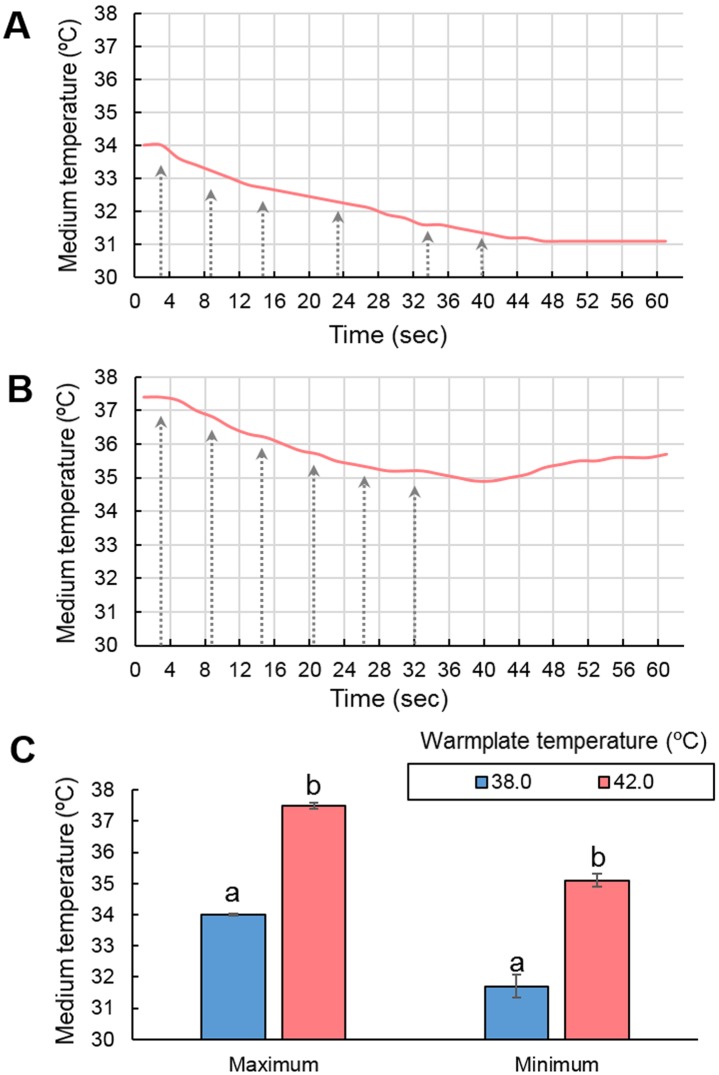
Changes of medium temperature during consecutive insertion of 6 vitrified microdrops (denoted with arrows) in a common 35 mm petri dish containing 2.5 ml of warming medium. A) a typical pattern of temperature changes on a 38°C warmplate; B) a typical pattern of temperature changes on a 42°C warmplate; C) mean ± SEM values of maximum and minimum temperatures recorded on 38°C and 42°C warmplates. Values with different superscripts (a, b) between treatment groups differ significantly (*P*<0.05) by student’s t-test.

Monitoring temperature changes during warming of vitrified microdrops revealed that both on a 38°C and 42°C warmplate, medium temperatures remained approximately 4°C below the actual temperature of the warmplate even before the start of warming procedure (Figure 2). The consecutive insertion of 6 vitrified microdrops resulted in a similar gradual decrease (2.3 and 2.4°C, respectively) of the warming medium in the dish (Figure 2 A and B). However, when warmplate temperature was set to 42°C the recorded maximum and minimum temperatures were significantly higher than those recorded on the 38°C -warmplate (Figure 2 C).

### Effect of pFF Supplementation in IVM Medium on Survival, Maturation, Fertilization and *In vitro* Development of Vitrified Porcine Oocytes

The percentages of survived oocytes after vitrification, warming and IVM did not differ between the pFF+ and pFF− groups (84.0%, and 83.5%, respectively). Also, the percentages of surviving oocytes reaching the M-II stage were similar between these groups (61.8% and 58.8%, respectively) (Table 3). After IVF of surviving oocytes, percentages of oocytes showing sperm penetration, male pronuclear formation, monospermy and normal fertilization did not differ significantly between the two groups (Table S2 in File S1). Furthermore, embryo development during culture appeared statistically similar between the two groups (Table 4). However, the total cell numbers in blastocysts showed a tendency to be lower in the pFF− group compared with the pFF+ group.

**Table 3:** Survival and nuclear maturation of vitrified oocytes after IVM for 44

pFF in IVM medium	Total	Live (% total)	Matured (with 1 PB)
+	531	446 (84.0±1.5)	271 (61.8±1.8)
–	541	454 (83.5±3.5)	266 (58.8±2.1)

Five replications were performed. Data are presented as means ± SEM.

IVM = in vitro maturation, pFF = porcine follicular fluid, PB = polar body.

No significant difference was detected between the treatment groups by student’s t-test.

**Table 4:** *In vitro* embryo development after IVF of vitrified oocytes matured in the presence or absence of pFF.

pFF in IVM medium	Total§	Cleaved (%)	Blastocyst (% total)			No. of cells/blastocyst
			day 5	day 6	day 7	
+	340	122 (36.0±1.3)	6 (1.8±0.3)	12 (3.5±0.3)	13 (3.8±0.4)	50.0±2.5
–	354	112 (32.5±3.0)	5 (1.4±0.1)	11 (3.1±0.1)	12 (3.4±0.4)	44.3±1.5*

Five replications were performed. Data are presented as means ± SEM.

No significant difference was detected between the treatment groups by student’s t-test.

*P = 0.053.

§ Live oocytes at 44 h IVM.

IVF = in vitro fertilization, Day 0 = the day of IVF.

### Transfer of Blastocysts Obtained from Vitrified–warmed Oocytes to Recipients

**Figure 3:**
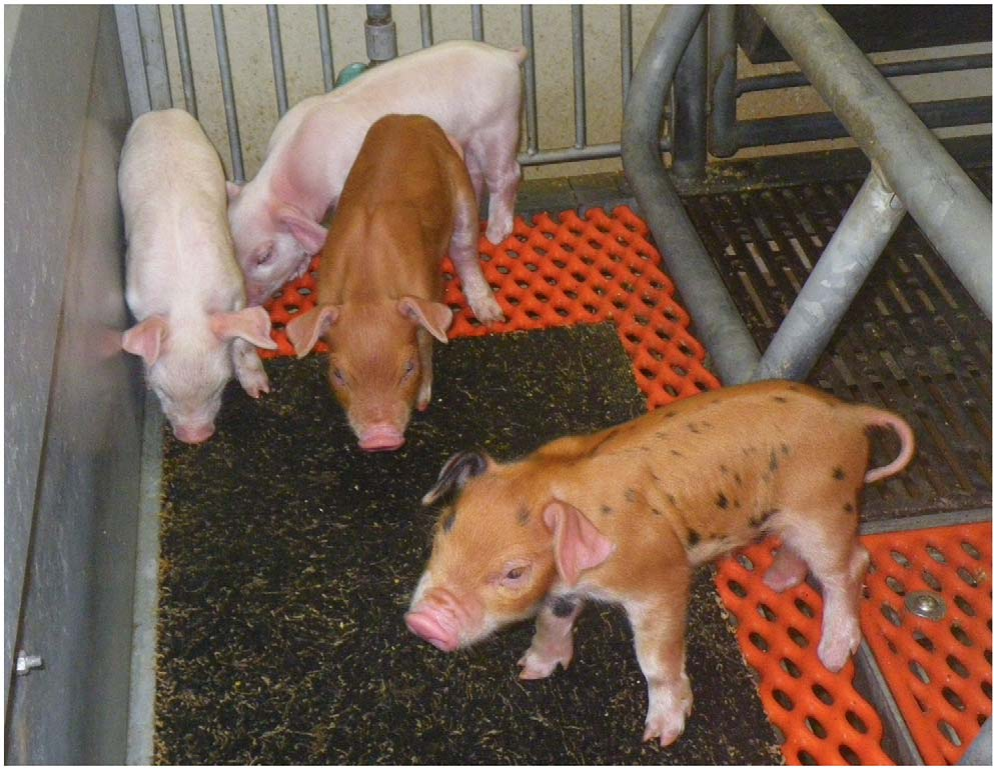
The first piglets obtained by the transfer of IVP blastocysts obtained from vitrified oocytes. The photograph was taken 5 days after delivery.

Transfer of vitrified–warmed and fertilized oocytes at the blastocyst stage into 4 recipients resulted in 4 pregnancies, which were all maintained until term. The pregnant recipients farrowed a total of 18 live piglets (Figure 3, Table 5). None of the piglets were stillborn. The piglets showed a little variation in weight at birth (Table 5). Both gestation period and body weight of piglets born were within expected ranges of normality.

**Table 5:** Production of piglets by the transfer of in

Recipient	pFF in IVM medium	Total Vitrifiedoocytes	No. embryostransferred	Gestation length(days)*	Total No. of piglets born (No. of live piglets)	Gender	Average body weight of piglets at birth (kg)$
#1	+	567	16	115	4 (4)	♂: 2/♀: 2	1.50±0.04
#2	+	1235	27	114	6 (6)	♂: 3/♀: 3	1.52±0.05
#3	–	954	17	117	5 (5)	♂: 3/♀: 2	1.34±0.19
#4	–	1018	13	115	3 (3)	♂: 2/♀: 1	1.37±0.04
Overall		3774	73		18 (18)	♂: 10/♀: 8	

*Day 0 = 2 days after hCG injection.

$ Data are presented as means ± SEM.

pFF = porcine follicular fluid, IVM = in vitro maturation.

## Discussion

Our results demonstrate that cumulus enclosed porcine oocytes could be successfully cryopreserved at the GV stage by SSV vitrification in large groups (approximately 100 oocytes/group) with high survival rates and without reducing their potential to undergo nuclear maturation and fertilization during IVM and IVF. Although the cryopreserved oocytes showed reduced developmental competence after IVM and IVF, approximately 5% of them developed to the blastocyst stage during IVC. Blastocyst formation in vitrified groups showed a slight but significant delay in vitrified groups. However, at the end of IVC (day 7), the quality of resultant blastocysts in terms of total cell numbers did not differ significantly between the vitrified and control groups (Table 2). Furthermore, transfer of day-5 blastocysts generated from vitrified oocytes resulted in the birth of live piglets. To our knowledge, these are the first piglets obtained from vitrified oocytes.

The success of piglet production by ET greatly depends on the number and quality of embryos transferred in each recipient. Low numbers and compromised quality of transferable embryos produced from vitrified oocytes are the most plausible reasons for the lacking success of piglet production using vitrified oocytes to date. The number of transferable embryos is determined by the capacity of the actual cryopreservation protocol (i.e. the number of oocytes preserved and utilized at one setting), the survival rate of the oocytes and their competence to undergo the processes of maturation, fertilization and embryo development after warming. The successful piglet production from vitrified oocytes using IVF and IVP in the present study can be attributed to the following factors; 1) the high capacity of the SSV vitrification method; 2) the high oocyte survival; 3) the normal competence of oocytes to undergo maturation and fertilization during IVM/IVF; and 4) the IVC and ET system that allowed the selection and transfer of high quality embryos into recipients. Our current vitrification protocol allows the vitrification and warming of oocytes in large groups (up to approximately 120 oocytes per setting). One vitrification setting includes the pre-treatment of a group of COCs with cytochalasin B (30 min), their equilibration (15 min) and vitrification (2×30 sec) and overlapping of settings is possible. Thus, approximately 300 COCs (3 settings) can be vitrified in 12–18 micro drops in total within 1.5 hours. The oocytes of one vitrification setting are preserved in 4–6 microdrops which are stored in a common cryotube and handled as one unit later during warming. The warming of one setting takes approximately 7 minutes in total. This high preservation capacity was associated with high survival and maturation rates. In the present study vitrification and warming of immature oocytes resulted in a 66.9% mean oocyte survival when the temperature of the warmplate was set at 38°C during warming. The process of warming includes the consecutive placement of 4–6 microdrops vitrified as one setting in a common warming dish (a 35 mm petri dish with 2.5 ml warming medium). In the present study, we measured medium temperature in the warming dish using a thermometer with a highly sensitive sensor. It revealed that, when the warming dish filled with 2.5 ml pre-warmed medium was kept on the warmplate heated to 38.0°C for several minutes, the mean temperature of the medium was 34.0°C. The warming process resulted in the gradual decrease of the temperature of the medium after the placement of each microdrop to an average minimum of 31.7°C (Figure 2). On the other hand, the mean temperature of the medium in the warming dish kept on a 42°C warmplate was 37.5°C, which was significantly higher than that on the 38°C warm plate. Similarly to the results using a 38°C warmplate, the medium temperature dropped approximately 2.4°C during the entire warming process, to an average minimum point of 35.1°C which was significantly higher than that measured on a 38°C warmplate. Suboptimal low temperatures of the warming medium reduce warming speed which in turn can reduce the percentage of live vitrified oocytes after warming [10], [11]. Accordingly, elevation of the temperature of the warmplate to 42°C during the first 2 minutes of warming prevented the excessive drop of medium temperature below 34.0°C which significantly increased the oocyte survival rate from 66.9% to 87.1%. These results demonstrate that optimization of the actual temperature in the proximity of oocytes during warming is a key for high survival rates after vitrification. Although surviving vitrified oocytes showed similar abilities to develop to the blastocyst stage after warming on 38°C and 42°C warmplates, higher survival rates on 42°C resulted in a significantly increased total blastocyst yield compared with warming on 38°C. In general, elevating temperatures to 42°C for much longer period such as several hours is known to be detrimental for porcine oocytes causing heat shock which manifests in cytoskeleton alterations, apoptosis and reduced developmental competence [22], [23]. However, our measurements revealed that, when the warming dish is kept on a 42°C warmplate, the actual medium temperature remained at the optimum zone which may not raise any concern for heat stress.

A further important advantage of the present vitrification protocol is that it did not reduce significantly the competence of GV stage oocytes to undergo nuclear maturation during IVM and subsequent fertilization during IVF. This result was in accordance with our previous studies [4], [6]. Our recent study revealed that after IVM of vitrified oocytes even the morphology of the cytoskeleton was similar to that of non-vitrified oocytes [4]. However, despite of their normal nuclear maturation and fertilization, vitrified oocytes still showed a significantly reduced competence to develop to the blastocyst stage during culture after IVF. It is generally observed that cryopreservation reduces the developmental ability of porcine oocytes to the blastocyst stage [2], [13]. The exact factors that cause the reduced competence remain unclear and require further research. The IVP procedure itself - which is essential for the utilization of vitrified porcine oocytes - predominantly limits blastocyst yields because of the high frequencies of polyspermy during IVF and the high sensitivity of porcine embryos to oxidative stress during IVC [24], [25]. In the present study, this situation was further worsened by the denudation of oocytes before IVF, which is known to reduce fertilization rates [17], [26] in porcine IVF systems. Even though we still persisted in denuding oocytes after IVM in all treatment groups (including the control) since this was the only way 1) to evaluate polar body extrusion and maintain the viability of oocytes (so they could be used subsequently) and 2) to rule out the possible effects of the potentially altered cumulus compartment on fertilization events in vitrified oocytes.

Despite of the reduced developmental rates, in agreement with previous results [4], [5], the blastocyst quality in terms of total cell numbers that developed from vitrified oocytes on day 7 was not significantly different from that developing from non-vitrified oocytes. The high quality of blastocysts obtained from vitrified oocytes is confirmed by the production of live offspring in this study. Regarding these results, it is clear that the high capacity, high survival rates and normal maturation/fertilization provided by the current cryopreservation protocol were the key factors to compensate the low developmental rates of vitrified oocytes in order to obtain adequate numbers of transferable embryos and to produce offspring from them.

The presence of follicular fluid in medium during oocyte maturation has been known to greatly improve the developmental rates of porcine IVM oocytes to the blastocyst stage during in vitro culture [27]–[29]. Furthermore, our previous research has revealed that, when NCSU-37 is used as a base medium for IVM, the presence of pFF during IVM is crucial for vitrified immature oocytes to maintain/regain their ability to complete nuclear maturation [13]. On the other hand, since pFF contains several unknown factors, such IVM medium is considered undefined and therefore it is undesirable for scientific purposes. Recently POM, a more complex chemically defined medium has been developed for porcine oocyte maturation by Yoshioka and colleagues [15] which supported the nuclear progression of vitrified immature porcine oocytes during IVM even without pFF supplementation [13]. In the present study, we investigated the efficacy of the POM medium used for IVM with and without pFF supplementation to facilitate fertilization and subsequent embryo development during IVF and IVC. There was no significant difference between vitrified oocytes matured in POM either with or without pFF supplementation in terms of nuclear maturation, fertilization and monospermy rates and in vitro embryo development. Although mean cell numbers in blastocysts appeared to be tendentiously reduced in the absence of pFF, the transfer of blastocyst generated from vitrified oocytes matured in a defined POM medium and cultured in a defined PZM-5 medium resulted in live piglets which proves the potential of this defined IVP system for the utilization of vitrified oocytes to generate live offspring.

The presence of pFF in IVM medium increases the degrees of cumulus expansion [30], [31] and the frequencies of total sperm penetration and polyspermy when cumulus-enclosed oocytes are used for IVF [15]. In accordance with these previous reports, we also observed higher grades of cumulus expansion during IVM in pFF-supplemented POM medium compared with IVM in defined POM medium (without pFF), irrespective of vitrification (data not shown). However, in the present study, oocytes were denuded before IVF. Therefore, it remains unclear if omitting pFF from the IVM medium has any effect on fertilization when cumulus-enclosed oocytes are subjected to IVF.

The success of piglet production by ET greatly depends on the quality of embryos selected for transfer. In the present study, blastocyst stage-embryos were selected for this purpose on day 5 which is the normal timing for blastulation of IVP porcine embryos in PZM-5 medium [15]. Early blastulation of embryos can be considered as a marker for high developmental competence as it is associated with high competence to implant and develop to term in hamsters [32], cattle [33] and humans [34], [35]. Our first experiment revealed, that irrespective of the warming temperature, the process of vitrification and warming caused a slight but significant delay of blastulation during embryo culture (Figure 1) which suggests that they have altered developmental competence to the blastocyst stage. The exact causes and consequences of it remain unknown. On the other hand, for ET, only embryos which showed clear signs of blastulation on day 5 were selected. This strict selection of early blastocysts might have been crucial for the success of piglet production in this study.

The problem of compromised developmental competence of oocytes after vitrification is a remaining problem that must be addressed in future experiments. Further research will be necessary to clarify the reasons for compromised developmental competence of vitrified oocytes and to develop methods to prevent or reduce these alterations. Combining the present protocol with alternative strategies to maintain developmental competence of oocytes such as by applying a sublethal stress treatment before preservation [36] may be a possible approach to improve the efficacy of our vitrification method.

In conclusion, adjusting the temperature of warmplate during warming to 42°C resulted in high survival rates of immature porcine oocytes cryopreserved in a large group by SSV. Surviving oocytes maintained their maturation and fertilization potential irrespective of the presence of pFF when POM medium was used for IVM. Despite of reduced embryo development, early developing blastocysts produced from vitrified oocytes could develop to term resulting in the first piglets produced from cryopreserved porcine oocytes.

## Supporting Information

File S1 **This file contains Figures S1 and Tables S1 and S2.**
(DOC)
