# Supplementary material for: Generation of Live Piglets from Cryopreserved Oocytes for the First Time Using a Defined System for In Vitro Embryo Production
Source: PLoS One. 2014 May 20;9(5):e97731. doi: 10.1371/journal.pone.0097731 (PMC4028240; doi:10.1371/journal.pone.0097731)
Supplement: File S1 — This file contains Figures S1 and Tables S1 and S2. (DOC) [file pone.0097731.s001.doc]

**Somfai - Supporting information**

| **Treatment groups** | **Hotplate temperature**  **at warming** | **No (%±SEM) of oocytes** | | | | |
| --- | --- | --- | --- | --- | --- | --- |
|  |  | **Total^§^** | **Penetrated (% total)** | **Normal* (% total)** | **MPN (% penetrated)** | **Monospermy (% penetrated)** |
| Control | – | 92 | 58 (61.6 ± 9.5) | 18 (19.3 ± 5.7) | 76 (77.7 ± 2.8) | 26 (47.0 ± 7.8) |
| Vitrified | 38 °C | 95 | 61 (64.1 ± 8.3) | 14 (14.7 ± 2.4) | 52 (84.2 ± 4.8) | 30 (51.0 ± 4.8) |
| Vitrified | 42 °C | 95 | 53 (55.5 ± 7.2) | 11 (11.6 ± 1.2) | 42 (79.6 ± 4.2) | 27 (52.2 ± 4.9) |

**Table S1. Fertilization status 10 h after IVF of control and vitrified live oocytes warmed at different temperatures and matured in POM supplemented with pFF.**

Five replications were performed. Data are presented as means ± SEM.

No significant difference was detected between the treatment groups by one-way ANOVA followed by Tukey’s multiple comparison test.

^§^ Live oocytes at 44 h of in vitro maturation.

*Normal fertilization status was characterized by the presence of 2 polar bodies, 1 male pronucleus and 1 female pronucleus.

IVM = in vitro maturation, IVF = in vitro fertilization, pFF = porcine follicular fluid, MPN = male pronucleus

**Table S2. Fertilization status 10 h after IVF of vitrified live oocytes matured in the presence or absence of pFF.**

| **pFF in IVM medium** | **No (%±SEM) of oocytes** | | | | |
| --- | --- | --- | --- | --- | --- |
|  | **Total^§^** | **Penetrated (% total)** | **Normal* (% total)** | **MPN (% penetrated)** | **Monospermy (% penetrated)** |
| + | 88 | 57 (63.8 ± 8.1) | 17 (19.3 ± 3.1) | 52 (88.1 ± 6.2) | 31 (56.7 ± 4.5) |
| – | 90 | 53 (59.6 ± 8.8) | 12 (13.0 ± 4.1) | 47 (85.6 ± 7.4) | 24 (48.7 ± 7.7) |

Five replications were performed. Data are presented as means ± SEM.

No significant difference was detected between the treatment groups by student`s t-test.

^§^ Live oocytes at 44 h of in vitro maturation.

*Normal fertilization status was characterized by the presence of 2 polar bodies, 1 male pronucleus and 1 female pronucleus.

IVM = in vitro maturation, IVF = in vitro fertilization, pFF = porcine follicular fluid, MPN = male pronucleus

**Figure S1. Blastocysts and early blastocysts on day 5 produced from vitrified oocytes before embryo transfer.** Scale bar represents 200 µm.
